# Describing hydrogen-bonded structures; topology graphs, nodal symbols and connectivity tables, exemplified by five polymorphs of each of sulfathiazole and sulfapyridine

**DOI:** 10.1186/s13065-014-0076-x

**Published:** 2015-01-21

**Authors:** Michael B Hursthouse, David S Hughes, Thomas Gelbrich, Terence L Threlfall

**Affiliations:** Chemistry, Faculty of Natural and Environmental Sciences, University of Southampton, Southampton, SO17 1BJ UK; Department of Chemistry, Faculty of Science, King Abdulaziz University, Jeddah, 21588 Saudi Arabia; Institute of Pharmacy, University of Innsbruck, Innrain 52, Innsbruck, 6020 Austria

## Abstract

**Background:**

Structural systematics is the comparison of sets of chemically related crystal structures with the aim to establish and describe relevant similarities and relationships. An important topic in this context is the comparison of hydrogen-bonded structures (HBSs) and their representation by suitable descriptors.

**Results:**

Three different description methods for HBSs are proposed, a graphical representation, a symbolic representation and connectivity tables. The most comprehensive description is provided by a modified graph of the underlying net topology of an HBS which contains information on the multiplicity of links, the directionality and chemical connectivity of hydrogen bonds and on symmetry relations. By contrast, the alternative symbolic representation is restricted to essential properties of an HBS, i.e. its dimensionality, topology type and selected connectivity characteristics of nodes. A comparison of their connectivity tables readily identifies differences and similarities between crystal structures with respect to the intermolecular interaction modes adopted by their functional groups. The application of these methods to the known polymorphs of sulfathiazole and sulfapyridine is demonstrated and it is shown that they enable the rationalisation of previously reported and intricate relationships.

**Conclusions:**

The proposed methods facilitate the comprehensive description of the most important relevant aspects of an HBS, including its chemical connectivity, net topology and symmetry characteristics, and they represent a new way to recognise similarities and relationships in organic crystal structures.

**Graphical Abstract:**
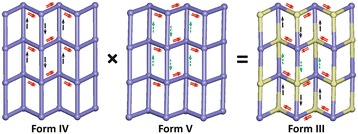
Graphical Representation of mixing of structures StzIV and StzV to give structure StzIII.

**Electronic supplementary material:**

The online version of this article (doi:10.1186/s13065-014-0076-x) contains supplementary material, which is available to authorized users.

## Background

In crystallographic studies, the structural systematics approach is used to increase our knowledge and understanding of the assembly of organic molecules into crystal structures [1-10]. Such investigations are carried out on polymorphs, solvates, salts and molecular complexes, in which a particular molecule can occur in different crystal structure environments, but also with families of compounds, whose molecular structures are very closely related, through small but systematic modifications to a parent molecule.

As the forces acting during the assembly of molecules into crystal structures are diverse, they should be considered in their entirety in any assessment. Consequently, the search for packing similarities, based only on geometrical considerations, has to be the cornerstone of any strategy for the comparison of groups of structures, and the XPac software [11] was developed in our laboratory for this purpose. However, structural patterns often reflect the presence of directed intermolecular interactions, exemplified by hydrogen bonding between conventional [12] donor and acceptor groups. The identification, description and comparison of such patterns could provide valuable pointers for progress in the area of crystal structure design and crystal growth. Even though geometrically similar structure patterns associated with hydrogen bonding are regularly identified as an integral part of an XPac study, the most fundamental property of a hydrogen-bonded structure (HBS) is its specific mode of intermolecular connections, and two molecular packing arrangements which agree in this characteristic are not necessarily also geometrically similar. Accordingly, a further strategy for identifying and describing structural similarities is required which enables the systematic comparison of different crystal structures with respect to their HBSs. Several useful methods for the description of certain aspects of an HBS have been proposed in the past, but none of these provides a comprehensive picture or is particularly suited for the structural systematics approach.

### Hydrogen-bonded structures – some considerations

Methodologies for describing networks in crystal structures of organic compounds which are based on intermolecular interactions have been frequently discussed over many years. Indeed, this is a sub-topic in an area of much wider scope, interest and activity, which also concerns the topology of network structures in elemental solid forms, through simple mixed inorganic solids − silicates, zeolites and the like, and, more recently metal-organic frameworks (MOFS). Palin and Powell [13] first described an organic crystal as a network with molecules as nodes, linked by H-bonds. Wells further explored this idea, initially in tandem with his descriptions of inorganic solid state structures [14] and subsequently in more focussed studies [15], and developed a classification scheme based on molecules as single points, with connecting H-bonds as lines. Kuleshova and Zorky [16] proposed a symbolic graphical description which is based on the essential unit of the underlying net of the HBS. The aforementioned authors introduced the descriptor ${G}_m^n(k)$, where the symbol *G* corresponds to the dimensionality of the HBS as either an island (i.e. finite cluster, I), chain (C), layer (L) or framework (F). The parameters *n* and *m* were originally defined by Wells [15], with *n* being the number of intermolecular H-bonds formed by a molecule and *m* the number of molecules to which the latter is joined, while *k* denotes the size of the essential ring of the net (for the whole crystal, the ratio between the number of H-bonds and the number of molecules is *n*/2).

The link between molecular networks and the classical infinite structures of inorganic mineral types became very clear when topologies of both types were compared, and the same network nomenclature was shown to be relevant for their classification [17]. In their 2005 monograph on networks in molecule based materials, Öhrstrom and Larson reviewed the terminology which is largely still in use today and gave a summary of the developmental thinking [18]. More recent work has focussed on enhanced software for analysing and producing graphical representations of networks, exemplified by the program TOPOS, developed by Blatov and collaborators [19,20] which is based on the Voronoi polyhedron partitioning approach to identify intermolecular contacts. The most recent developments have included capabilities to represent packing geometries also for molecular crystals which are not necessarily dependent on hydrogen bonding [21-23]. Here, the connection of molecular nodes, based on positive Voronoi contact is used to define the type of net.

A method for the representation of the more local characteristics of an HBS was proposed by Etter [24,25], who implicitly considered the actual chemistry behind the H-bonds – that is, which functional groups are bonded to which others? This led to a number of papers, also by other authors (e.g. Bernstein [26,27]), in which a graph-set approach was used to describe HBSs. This methodology has been widely adopted, in particular for the description of sub-components of HBSs, such as rings and chains. Due to its very specific nature this nomenclature has somewhat limited value for comparisons, e.g. the symbol ${\mathrm{R}}_2^2(8)$. describes a ring which is closed by two pairs of functional groups, and the 8 identifies the total number of atoms in the ring. By contrast, in the area of “nodal networks” the size of the ring is not significant, since topologically, these atoms are mainly spacers in a system in which a node (molecule) is linked to another node via two connectors, usually of the donor-acceptor type. Analogous molecules whose donor-acceptor connectors are separated by a different number of atoms may nevertheless form HBSs of the same topology.

In 1997 Desiraju [28] revisited some of the ideas quoted above, and also the work of Robertson [29], including the use of nodes and networks to describe packing and H-bonding in crystal structures, and suggested that the node connections were of greater significance than the nodes themselves. The possibilities offered by this approach and other methods cited above were subsequently explored by one of us [30]. The aim of the present work is the definition of a set of detailed, informative and useful descriptors for comparing HBSs, which answer to the questions listed below.

1. For a molecule involved in hydrogen bonding, which donor(s) are connected to which acceptor(s)?
2. What are the symmetry relationships between connected molecules?
3. What is/are the most informative way/s to represent the type and topology of the resulting array of connected molecules?

First, three different description methods for HBSs (graphical representation, symbolic representation and connectivity table) will be described. These methods will then be applied to the polymorphs of two closely related chemical compounds, sulfathiazole and sulfapyridine. The results obtained will be discussed in the context of both previous studies and alternative HBS description methods.

## Results

### Methods for the representation of an HBS

#### a) Graphical representation

Conventional hydrogen bonds [12], D − **H**∙∙∙**A**, are reliably formed between molecules with suitable functional groups that can serve as H-bond donors (D) and acceptors (**A**). In general, different sets of **H**/**A** combinations are possible, depending on the number of hydrogen atoms (**H**) that can be donated and the number of available acceptor sites. Each set of **H**/**A** combinations can lead to a variety of distinct HBSs, which are either finite (islands) or periodic in 1, 2 or 3 dimensions (chains, layers, frameworks). A suitable representation method should convey a maximum of information about an individual HBS and, at the same time, enable a comparison with other HBSs that are formed by the same molecule or by closely related molecules.

The underlying topology of an HBS is described by a net composed of nodes representing molecules and links representing intermolecular connections by D − **H**∙∙∙**A** bonds. Using the TOPOS software [31,32], a diagram of the net is readily obtained and its topology can be determined. The type of the net is denoted by the three-letter RCSR (Reticular Chemistry Structure Resource) symbol [33] or in case of a novel topology its point symbol [34] can be used instead. The topological net of an HBS exhibits the following additional and important characteristics:

1. it usually contains more than one crystallographically independent type of link;
2. a link can represent a one-point or multiple-point connection, i.e. two molecules are connected to one another by a single D − **H**∙∙∙**A** interaction or by multiple H-bonds;
3. a link between two chemically identical molecules can be associated with a crystallographic symmetry operation; in the case of a Z’ > 1 structure, the two H-bonded molecules can display a handedness relationship and possibly also a local symmetry or a pseudo-symmetry relationship;
4. the H-bonds which define the links possess a chemical identity, i.e. links are associated with specific **H**/**A** combinations;
5. each H-bond possesses directionality, i.e. **H** → **A**.

Therefore, a comprehensive representation of an HBS can be achieved with a modified diagram of the topological net containing the following additional features:

1. the RCSR symbol or the point symbol of the net;
2. crystallographically independent molecules are represented as nodes of different colour;
3. individual H-bonds are indicated by arrows (**H** → **A**) placed next to a link;
4. the underlying **H**/**A** combination(s) and a symbol for the associated symmetry element (or handedness relationship) are given for each link in the legend of the diagram.

Crystallographic symmetry elements are indicated by their printed symbols as defined in the International Tables of Crystallography [35]. Molecular conformations are relevant when polymorphs are compared, specifically the possible occurrence of molecular chirality. The latter can be either real, or conformational, i.e. constrained as a result of conformational restrictions, or, when fundamentally achiral molecules adopt rigid conformations when “frozen” in the solid state “pseudo-chirality”. Although pseudo-chirality is generally of no importance chemically, it is of considerable importance in crystal structure pattern descriptions. For a Z' = 1 structure, this type of conformational relationship is inherent in the crystallographic symmetry elements. For connections between chemically identical but crystallographically distinct molecules, a plus symbol (+) indicates that the latter have the same handedness and a minus (−) denotes that they are of the opposite handedness. Alternatively, the relevant symbols for known (local) pseudo-symmetry elements, enclosed in brackets, may be given. A cross (×) is used if no such relationship can be identified, in particular for connections between chemically distinct molecules.

#### b) HBS symbols / nodal symbols

The graphical representation provides the most comprehensive information about an HBS, but it may also be useful to encode just its most essential characteristics in a descriptor of the composition

$$ D{\left\{{n}_m\right\}}_1\cdot {\left\{{n}_m\right\}}_2\cdots \cdot {\left\{{n}_m\right\}}_p\left[T\right], $$

where *D* is a dimensionality symbol (C = chain, L = layer or F = framework), *n* the number of intermolecular H-bonds of a molecule, *m* the number of neighbours to which the latter is joined and *p* is the number of crystallographically independent molecules in the HBS. The expression {*n*_m_}_*i*_ denotes the connectivity symbol *n*_m_ for the *i*-th molecule (node) (*i* = 1, 2… *p*). *T* is a topology identifier of the net consisting of its point symbol [34], followed by the three-letter RCSR symbol [33] (if available), for example 4^2^.4^8^-**pts**, or another common name for the net.^a^

Both the dimensionality (*D*) of the HBS and the number of connected neighbours per molecule (*m*) are given explicitly as a matter of convenience, even though these parameters can also be deduced from the net topology type (*T*).

In an extended version, this descriptor is followed by a colon symbol and the symmetry information for the links of each of the *i* = 1, 2… *p* crystallographically independent molecules, enclosed in square brackets,

$$ D{\left\{{n}_m\right\}}_1\cdot {\left\{{n}_m\right\}}_2\cdots {\left\{{n}_m\right\}}_p\left[T\right]:{\left[{o}_1,\cdot {o}_2\cdots \cdot {o}_m\right]}_1\cdot {\left[{o}_1\cdot {o}_2\cdots \cdot {o}_m\right]}_2\cdots {\left[{o}_1\cdot {o}_2\cdots \cdot {o}_m\right]}_p, $$

where *o*_j_ is the relationship symbol for the symmetry or handedness relationship (see above) associated with the link to the *j*-th neighbour (*j* = 1, 2, …*m*). For links with multiple-point connections, an additional superscript Roman numeral indicates the number of H-bonds. [*o*_1_ ⋅ *o*_2_ ⋯ ⋅ *o*_m_]_i_ is the nodal symbol for the *i*-th molecule (with *i* = 1, 2… *p*) containing the symmetry symbols for its *m* links which are separated by dots and enclosed in square brackets.

#### c) Connectivity table

Connectivity tables are intended to facilitate the detailed analysis of the various **H**/**A** combinations which are formed by the comparable sets of functional groups. The table for a given HBS is generated by arranging all the potential H-bond donor sites (**H1**, **H2**…) in rows and the potential acceptor sites (**A1**, **A2**…) in columns. The order within both these sets follows a predefined specific assignment scheme used for the entire crystal structure series under investigation. The **A** and **H** sets for crystallographically distinct molecules are arranged in sequential order (e.g. **A1**, **A2**,…**A1'**, **A2'**,…**A1''**, **A2''**,…). The boxes within a connectivity table generated in this way represent all possible **H**/**A** combinations. Those boxes (**H**/**A** combinations) which correspond to an observed intermolecular D − **H**∙∙∙**A** bond contain the printed symbol for the corresponding symmetry element or handedness relationship (see above). Interactions between chemically distinct molecules are denoted by a cross (×) and intramolecular H-bonds by the symbol *S* (“self”).

The involvement of an **H** or **A** site in a certain number of H-bond interactions results in the same number of entries in the corresponding row (**H**) or column (**A**). For a given molecule the sum of all entries (except for the symbol *S*) in the rows associated with, plus the sum of all entries in the corresponding columns equals the number *n* of its intermolecular H-bonds. The analysis of a set of H-bond connectivity tables gives an overview of viable **H**/**A** combinations and shows preferred **H**/**A** pairings. However it is not possible to draw conclusions about the topology type of an HBS solely from the information contained in its connectivity table. A rather different type of matrix known as NIPMAT (nonbonded interaction pattern matrix) [36] for the rationalisation of all intermolecular interactions was previously proposed by Rowland [37].

### Application to polymorphs of sulfathiazole

#### a) General

**Figure 1:**
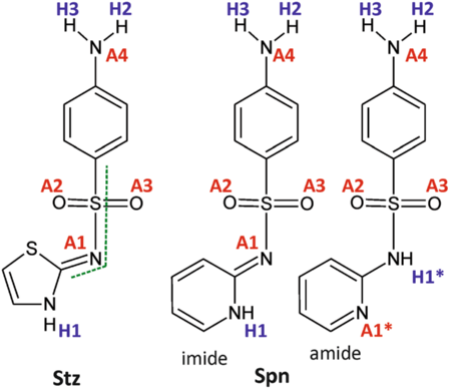
**Definition of D and A sites in the molecules of sulfathiazole (Stz; broken line: torsion angle C − N − S − C) and sulfapyridine (Spn).**

Sulfathiazole (Stz), 4-amino-N-(1,3-thiazol-2-yl)benzenesulfonamide, is a classical polymorphic compound with known crystal structures of five polymorphs (denoted **Stz**-**I**, **Stz**-**II**, **Stz**-**III**, **Stz**-**IV** and **Stz**-**V**, in accordance with the pharmaceutical nomenclature [38]; Additional file 1: Table S1) and more than 100 solvates [38-41]. Blagden et al. described the HBSs of four polymorphs [39] using Etter’s graph set methodology [24], and the packing relationships of five Stz forms were previously investigated by us [38]. The Stz molecule contains three D − **H** and four **A** sites (Figure 1) which can engage in classical D − **H**∙∙∙**A** interactions. The Stz polymorphs family provide a very good example to demonstrate the advantages of our approach because their HBSs are among the most complex and diverse found in small organic molecules.

#### b) Definition of matching H and A sites

Sulfathiazole is an example of a pseudo-chiral system and indeed Blagden et al. [39] first coined the term pseudo-chirality in their analysis of Stz polymorphs. This pseudo-chirality originates from the freezing-in of the conformation adopted for the S-sulfonamido single bond, characterised by the corresponding torsion angle C − N − S − C. Moreover, all the known Stz polymorphs contain the imide tautomer with the proton on the ring nitrogen atom. The **A** and **H** sites were assigned according to the following rules (Figure 1):

1. **A1** is the imido N atom;
2. **A2** is the sulfonyl O atom associated with the largest absolute value of the torsion angle C − N − S − O and **A3** is the other sulfonyl O atom;
3. **A4** is the aniline N atom;
4. **H1** is the H atom of the amido nitrogen NH group;
5. **H2** is the H atom of the aniline NH_2_ group which gives the largest absolute value of the pseudo-torsion angle **A2** − S∙∙∙**A4** − H, and **H3** is the other H atom of the same group.

Details of this assignment and the relevant torsion angles are listed in Additional file 1: Tables S2 and S3. Geometrical parameters for D − **H**∙∙∙**A** bonds are given in Additional file 1: Tables S3 and S6 − S9. The order in which the polymorphs are discussed in the next sections (**Stz**-**IV**, −**V**, −**III**, −**II**, −**I**) follows the increasing complexity of their HBSs.

#### c) Polymorph Stz-IV

**Figure 2:**
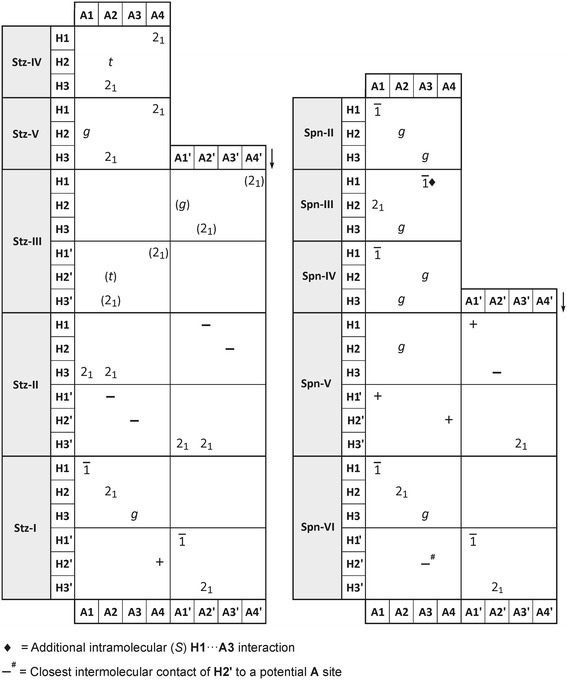
**Connectivity tables for the D − H∙∙∙A interactions in polymorphic forms of sulfathiazole (Stz) and sulfapyridine (Spn).**

The polymorph **IV** has the monoclinic space group *P*2_1_/c and its asymmetric unit contains one molecule. Two parallel hydrogen bonds link neighbouring **Stz** molecules into a chain with two-fold screw symmetry. In this chain, each molecule is bonded via its amido group to the aniline N atom of a neighbouring molecule (**H1**∙∙∙**A4**) and also via the aniline **H3** site to the sulfonyl site **A2** (**H3**∙∙∙**A2**) of the same molecule. Additionally it forms **H2**∙∙∙**A2** bonds to two other molecules to which it is related by translations along the *a* axis. These latter interactions involve the second aniline H atom (**H2**) and again the sulfonyl O atom **A2**. Neither the imido N atom **A1** nor the sulfonyl site **A3** are used, while the sulfonyl site **A2** is employed in two H-bonds, as can be seen from the connectivity table in Figure 2.

**Figure 3:**
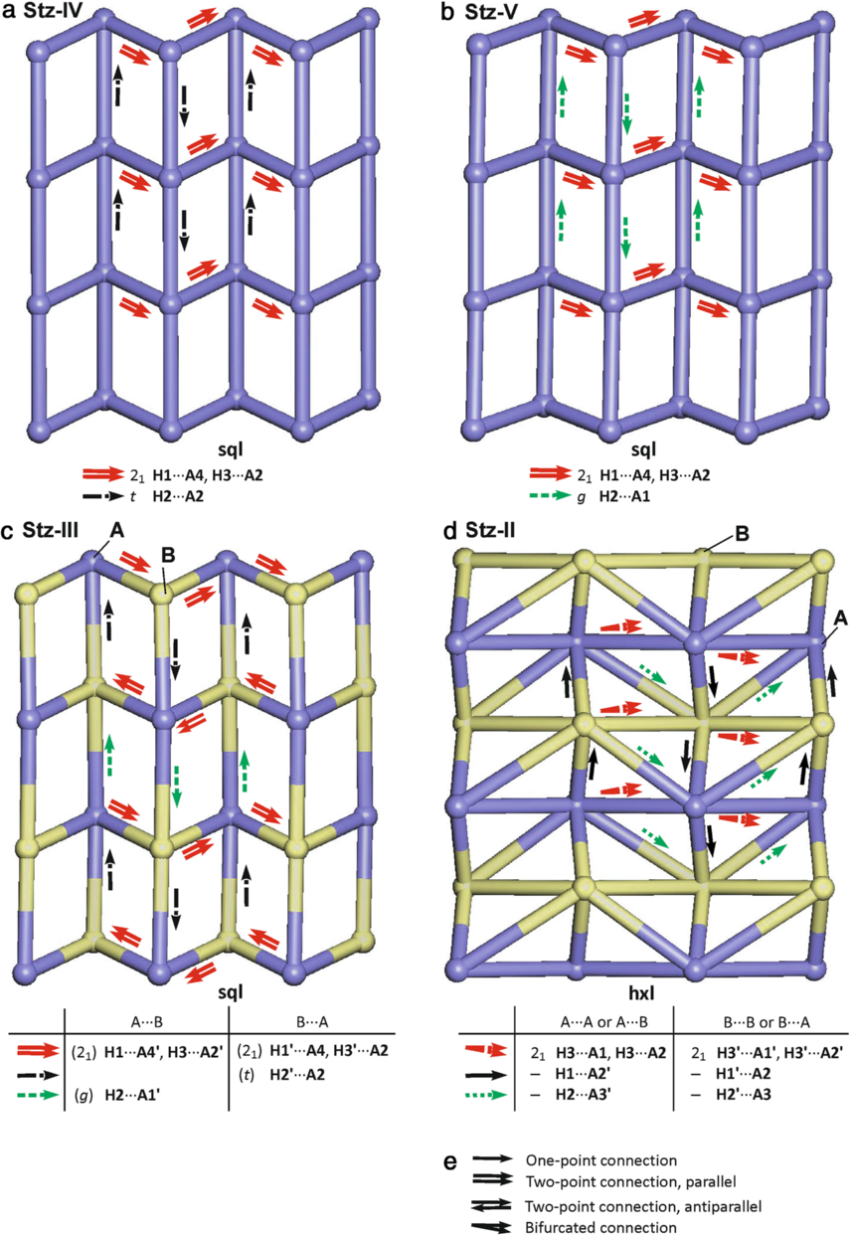
**Topology graphs for the hydrogen bonded layer structures of the forms a) IV, b) V, c) III and d) II of sulfathiazole (Stz) and e) different arrow types used for the representation of one-point and two-point connections.**

Altogether, each molecule is engaged in six hydrogen bonds which connect to four neighbouring molecules, resulting in a layer structure with **sql** topology which lies parallel to (001) (Figure 3a) and whose symbol is L6_4_[4^4^.6^2^-**sql**]. The extended symbol L6_4_[4^4^.6^2^-**sql**]:[2_1_^II^.*t*.2_1_^II^.*t*] indicates the presence of two-point H-bond connections along the screw axis (denoted by parallel arrows in Figure 3a). Due to the symmetry elements involved (2_1_, *t*) all molecules of a single H-bonded layer are of the same handedness while neighbouring layers are related by an inversion operation.

#### d) Polymorph Stz-V

The crystal structure of form **V** has the space group symmetry *P*2_1_/*n* and contains one independent molecule. Analogous to **Stz**-**IV**, parallel two-point connections are formed as a result of **H1**∙∙∙**A4** and **H3**∙∙∙**A2** interactions to give a chain of H-bonded molecules with two-fold screw symmetry. Additionally, molecules that are related by a glide-reflection operation are H-bonded via the **H2** position of the aniline NH_2_ group of one molecule and the **A1** sulfonyl O site of the other (**H2**∙∙∙**A1**).

Altogether, each molecule is connected to four neighbours via six hydrogen bonds, resulting in an **sql** net parallel to (101) (Figure 3b), which has the same symbol, L6_4_[4^4^.6^2^-**sql**], as that net of **Stz**-**IV**. However, the long symbol for **Stl**-**V**, L6_4_[4^4^.6^2^-**sql**]:[2_1_^II^.*g*.2_1_^II^.*g*], accounts for the fundamental difference in the symmetry of the links compared to form **IV**. Moreover, the connectivity table for **Stz**-**V** in Figure 2 shows that all available H-bond donor sites, except for **A3**, are employed once, whereas in **Stl**-**IV** the **A2** function accepts two hydrogen bonds while neither **A1** or **A3** are engaged in a D − **H**∙∙∙**A** interaction. The two-point connections in the net of form **V** link molecules of the same handedness, and the one-point connections (glide-reflection symmetry) link molecules which have the opposite handedness.

#### e) Polymorph Stz-III

The crystal structure of form **III** has the space group symmetry *P*2_1_/*c* and contains two independent molecules, denoted A and B. Each A molecule donates two hydrogen bonds of the **H1**∙∙∙**A4'** and **H3**∙∙∙**A2'** types to molecule B and in turn it accepts two analogous hydrogen bonds from a second B-molecule, i.e. **H1'**∙∙∙**A4** and **H3'**∙∙∙**A2**. Resulting from these parallel two-point connections, alternating A and B molecules of the same handedness are linked into an H-bonded chain parallel to [010]. Indeed, it was shown that this chain possesses a non-crystallographic 2_1_ symmetry [38]. The **H2** site of the aniline NH_2_ group in molecule A is bonded to the sulfonyl O site **A2** of a B molecule of the opposite handedness (**H2**∙∙∙**A1'**), and the A and B molecules involved in this particular interaction are related by a local glide-reflection operation [38]. The **H2'** site of molecule B is bonded to the sulfonyl O site **A2** of an A-type molecule which is related to this B molecule by a local translation operation [38], i.e. both are of the same handedness.

Altogether, the D − **H**∙∙∙**A** interactions result in a **sql** net parallel to (10_._$\overline{2}$) in which the two molecule types are arranged in an alternating fashion along the links (Figure 3c). This net is uninodal, but the A and B sites differ in the local (glide-reflection plane or translation) symmetry element (and therefore in the kind of pseudo-chirality relationship) associated with two of their hydrogen bonds. Simultaneously, they differ in the sulfonyl-O acceptor (A: **A2** vs. B: **A1**) that is bonded to the **H2'** / **H2** site of a molecule of the other type. The short symbol of the H-bonded layer structure, L6_4_.6_4_[4^4^.6^2^-**sql**], reflects the fact that it is composed of two independent molecules which are both connected to four neighbours via six H-bond interactions. The long symbol is L6_4_.6_4_[4^4^.6^2^-**sql**]:[(2_1_)^II^.(*g*).(2_1_)^II^.(*t*)][(2_1_)^II^.(*t*).(2_1_)^II^.(*g*)] if local symmetry elements are considered or alternatively L6_4_.6_4_[4^4^.6^2^-**sql**]:[+^II^. + . + ^II^.+][+^II^. − . + ^II^.−] if only pseudo-chirality relationships are considered.

#### f) Polymorph Stz-II

The crystal structure of polymorph **II** has the space group symmetry *P*2_1_/*n* and contains two independent molecules, A and B. The **H3** site in the NH_2_ group of molecule A is bonded to the imido nitrogen site **A1** and additionally to the sulfonyl-O site **A2** of a second A molecule (**H3**∙∙∙**A1** and **H3**∙∙∙**A2**). This bifurcated two-point connection results in a chain of H-bonded A molecules which are arranged around a two-fold screw axis, and an analogous chain is formed by B molecules on the basis of **H3'**∙∙∙**A1'** and **H3'**∙∙∙**A2'** interactions. Additionally, each A molecule is connected to four B molecules of the opposite handedness via two pairs of analogous interactions involving the thiazole NH group (**H1**) and the NH_2_ group (**H2**) as the donor groups and the sulfonyl sites **A2** and **A3** as acceptor sites, i.e. A∙∙∙B: **H1**∙∙∙**A2'**, **H2**∙∙∙**A3'** and B∙∙∙A: **H1'**∙∙∙**A2**, **H2'**∙∙∙**A3**). From the connectivity table (Figure 2) and topology graph (Figure 3d) it can be seen that the A and B molecules display the same H-bond connectivity. Each molecule serves as a six-connected node within an **hxl** net, which lies parallel to (001). This layer structure contains alternating H-bonded chains propagating along [010], which are homochiral and composed exclusively of either A and B molecules. Each such chain is connected to two neighbouring chains of molecules of the other type which are of the opposite handedness. This HBS is described by the symbol L8_6_.8_6_[3^6^.4^6^.5^3^-**hxl**] as both types of molecule are involved in eight hydrogen bonds to six neighbours. The equivalence of the A and B molecules is also indicated by the long symbol L8_6_.8_6_[3^6^.4^6^.5^3^-**hxl**]:[2_1_^II^. − .2_1_^II^. − . − .−][2_1_^II^. − .2_1_^II^. − . − .−].

#### g) Polymorph Stz-I

**Figure 4:**
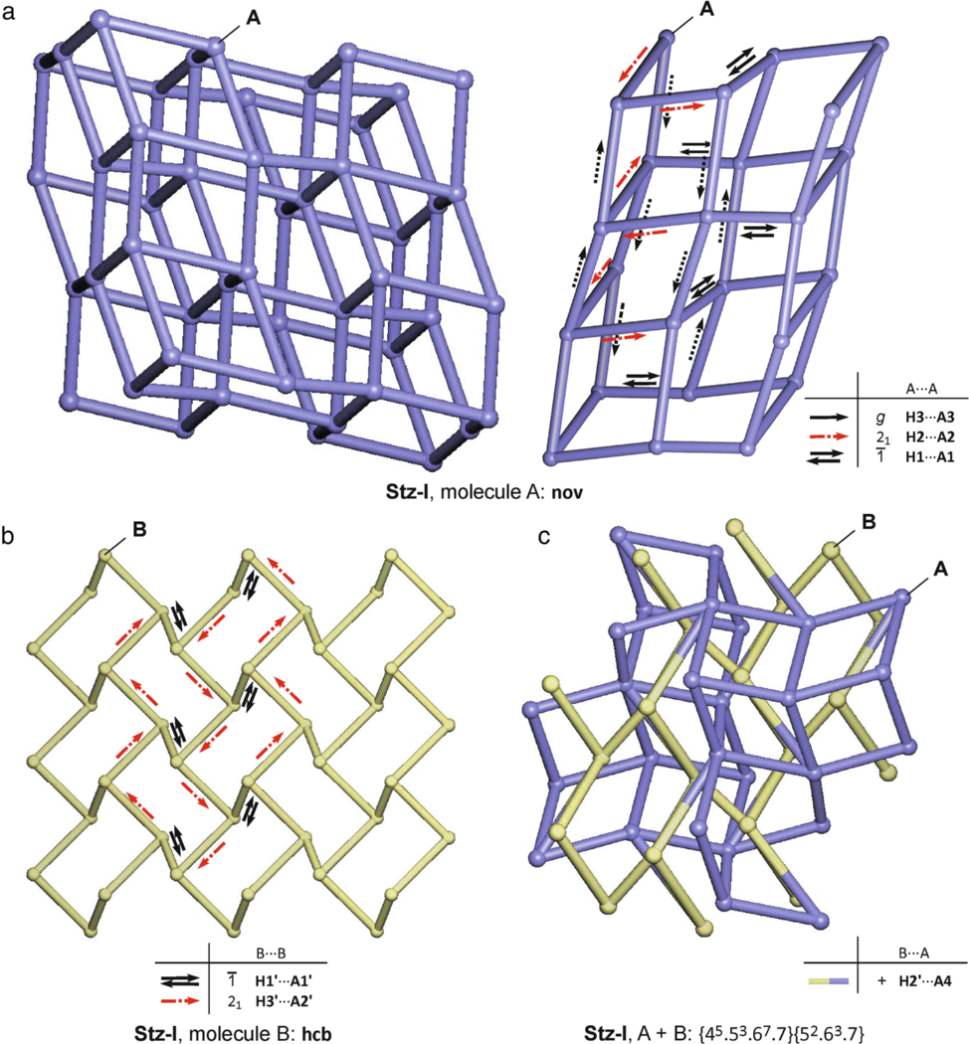
**Topology graphs for the hydrogen bonded structures of form I of sulfathiazole (Stz), showing separately a) the nov framework formed by molecules of type A, b) the hcb net formed by molecules of type B and then c) the framework of connected A and B molecules.**

**Stz**-**I** crystallises in the space group *P*2_1_/c with two independent molecules. Type-A molecules are connected to one another via three hydrogen bonds, **H1**∙∙∙**A1**, **H2**∙∙∙**A2** and **H3**∙∙∙**A3**. The first of these interactions involves the NH groups and imido N atoms of two molecules related by inversion symmetry and results in an antiparallel two-point connection. The other two interactions are formed between NH_2_ groups as the donor and sulfonyl O atoms as the acceptor sites, in one case via a screw operation and in the other via a glide-reflection operation. Altogether, each A molecule is connected to five other A molecules via six H-bonds, giving a 4^4^.6^6^-**nov** framework [42] with five-connected nodes (Figure 4a). Therefore, the isolated H-bonded structure of A-type molecules has the symbol F6_5_[4^4^.6^6^-**nov**]:[*g*.2_1_.*g*.2_1_.${\overline{1}}^{\mathrm{II}}$] (short: F6_5_[4^4^.6^6^-**nov**]).

The H-bonded B molecules form a separate layer structure and serve as three-connected nodes in a honeycomb-type (**hcb**) net (Figure 4b). This layer lies parallel to (100). Analogous to the framework of A molecules, it contains centrosymmetric units with antiparallel two-point H-bond connections of the **H1'**∙∙∙**A1'** type. Neighbouring B molecules are **H3'**∙∙∙**A2'** linked via their NH_2_ and sulfonyl groups so that chains with a two-fold screw symmetry are generated. Therefore, the symbol of the H-bonded structure of B molecules has the symbol L4_3_[6^3^-**hcb**]:[2_1_.${\overline{1}}^{\mathrm{II}}$.2_1_] (short: L4_3_[6^3^-**hcb**]).

The interpenetration of the **nov** framework (A) by a single **hcb** layer (B) structure is depicted in Figure 4b, and the two nets are linked by an **H2'**∙∙∙**A4** bond in which the NH_2_ groups of A and B molecules of the same handedness serve as the H-bond donor and acceptor site, respectively. The resulting A + B framework contains an equal number of six-connected and four-connected nodes and has the point symbol (4^4^.5^3^.6^7^.7)(5^2^.6^4^). Therefore, the long symbol for the complete H-bonded structure is F7_6_.5_4_[(4^4^.5^3^.6^7^.7)(5^2^.6^4^)]:[*g*.21.*g*.2_1_.${\overline{1}}^{\mathrm{II}}$.+][2_1_.${\overline{1}}^{\mathrm{II}}$.2_1._ +].

#### h) Relationship between Stz-I and Spn-VI

Sulfapyridine (4-amino-*N*-pyridin-2-ylbenzenesulfonamide; **Spn**) is a structural analogue of **Stz**, in which the thiazole unit is replaced by a pyridine ring (Figure 1). Crucially, the molecules of these two compounds contain matching functional groups for hydrogen bonds. The imide tautomer is present in all known solid forms of **Stz** and **Spn**, except for **Spn**-**VI** (space group *P*2_1_/*n*) which contains imide (A) as well as amide molecules (B). The position H1'* of the amido group and A1'* of the pyridine ring in the amide tautomer correspond to **H1** and **A1**, respectively, in the imide. In **Stz**-**I** and **Spn**-**VI,** both molecule types form centrosymmetric two-point H-bond connections of the type **H1**∙∙∙**A1** (imide) / H1'*∙∙∙A1'* (amide). The tautomeric form of the two H-bonded molecules determines the H position in the N − H∙∙∙N interaction of the resulting H-bonded dimer but does not alter the overall geometry of the dimer.

**Spn**-**VI** and **Stz**-**I** agree in the complete set of H-bond interactions between their respective type-A molecules, which result in a **nov** net (Figure 4a). The H-bond interactions between type-B molecules which generate the **hcb** net (Figure 4b) are also the same in **Spn**-**VI** and **Stz**-**I**. Therefore, the separate H-bonded A and B nets of **Spn**-**VI** have the same symbols as their counterparts in **Stz**-**I** (Table 1) and the connectivity tables for **Stz**-**I** and **Spn**-**VI** (Figure 2) agree in their upper left and lower right quadrants (A∙∙∙A and B∙∙∙B bonding).

**Table 1:** **Constituents of the HBS symbols for polymorphs of sulfathiazole (Stz) and sulfapyridine (Spn)**

**HBS**	**Topology**		**Node type 1**		**Node type 2**	
	***D***	***T***	***n***_***m***_	***o***_**1**_ **.** ***o***_**2**_ **….** ***o***_***m***_	***n***_***m***_	***o***_**1**_ **.** ***o***_**2**_ **….** ***o***_***m***_
**Stz**-**I**	F	(4^4^.5^3^.6^7^.7)(5^2^.6^3^.7)	7_6_	*g*.2_1._ *g*.2_1._ ${\overline{1}}^{\mathrm{II}}$_._+	5_4_	2_1_.${\overline{1}}^{\mathrm{II}}$_._2_1._+
**Stz**-**II**	L	3^6^.4^6^.5^3^-**hxl**	8_6_	2_1_^II^. − .2_1_^II^. − . − .−	8_6_	2_1_^II^. − .2_1_^II^. − . − .−
**Stz**-**III**	L	4^4^.6^2^-**sql**	6_4_	(2_1_)^II^.(*g*).(2_1_)^II^.(*t*)	6_4_	(2_1_)^II^.(*t*).(2_1_)^II^.(*g*)
**Stz**-**IV**	L	4^4^.6^2^-**sql**	6_4_	2_1_^II^.*t*.2_1_^II^.*t*		
**Stz**-**V**	L	4^4^.6^2^-**sql**	6_4_	2_1_^II^.*g*.2_1_^II^.*g*		
**Spn**-**II**	F	4^4^.6^6^-**sqp** ^a^	6_5_	${\overline{1}}^{\mathrm{II}}$.*g.g.g.g*		
**Spn**-**III**	L	3^3^.4^3^.5^4^-**tts**	6_5_	${\overline{1}}^{\mathrm{II}}$.2_1_.*g.*2_1_ *.g*		
**Spn**-**IV**	L	4^8^.6^2^-**SnS** ^a^	6_5_	${\overline{1}}^{\mathrm{II}}$.*g.g.g.g*		
**Spn**-**V**	F	(5^5^.6^4^.7)(5^6^.6^3^.7)^b^	6_5_	+ ^II^.*g*. − .*g*.+	6_5_	+ ^II^.2_1_. −.2_1_.+
**Spn**-**VI (A)**	F	4^4^.6^6^-**nov** ^c^	6_5_	*g*.2_1_.*g*.2_1_.${\overline{1}}^{\mathrm{II}}$		
**Spn**-**VI (B)** ^d^	L	6^3^-**hcb**	4_3_	2_1_.${\overline{1}}^{\mathrm{II}}$.2_1_		

^a^See ref. [43].

^b^SP 2-periodic net (4,4)Ia.

^c^See ref. [42].

^d^The A framework is interpenetrated by B layers.

These results are consistent with the previously reported 3D packing similarity of **Spn**-**VI** and **Stz**-**I** [44], which also implies a similar mode of interpenetration of the **nov**-type framework by **hcb** layers. This relationship was confirmed by an XPac comparison, which gave a dissimilarity index of *x* = 12.7 and distance parameter of *d* = 0.66 Å (for details, see section 4.2 of the Additional file 1), consistent with geometric deviations due to the relatively large difference in molecular shape between **Stz** and **Spn**.

**Figure 5:**
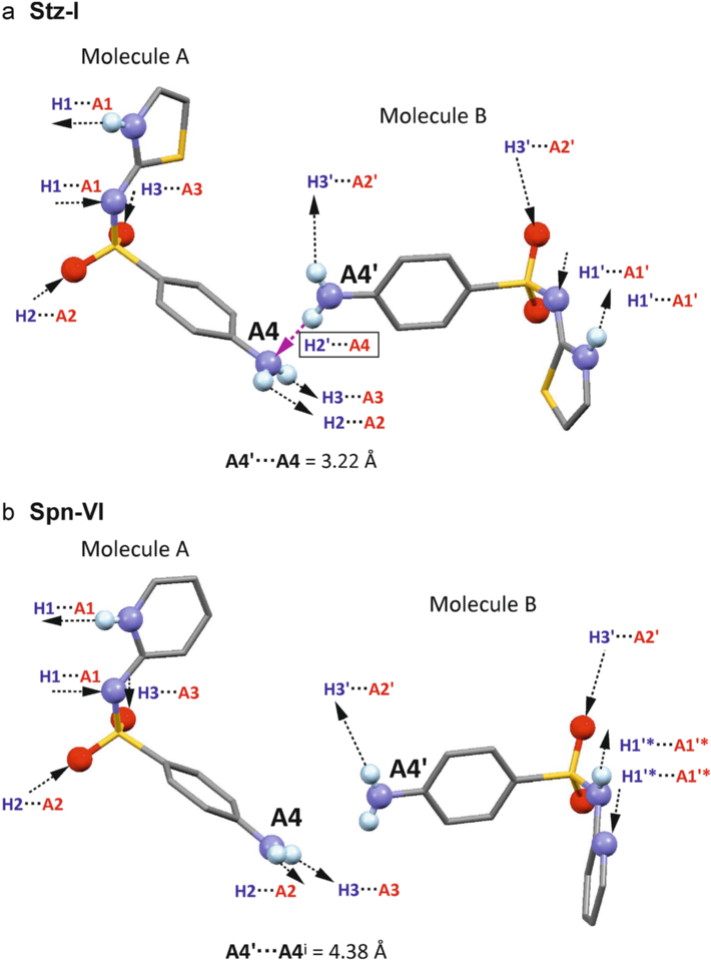
**Matching geometrical arrangements in the isostructural forms Stz-I and Spn-VI. (a)** Stz-I: A- and B-type molecules, connected by an H2'∙∙∙A4 bond, which serves as the only link between the nov **(A)** and hcb **(B)** nets. **(b)** Spn-VI: A larger separation between **A** and **B** molecules results in the absence of an H2'∙∙∙A4 connection between the nov and hcb nets. Symmetry operation (i) 3/2 - x, 1/2 + y, 1/2 - z.

A fundamental difference between **Stz**-**I** and **Spn**-**VI** concerns the **H2'**∙∙∙**A4** link between the **hcb** and **nov** nets in **Stz**-**I** (with H∙∙∙N and N∙∙∙N distances of 2.29 and 3.22 Å, respectively, between A and B molecules of the same handedness; see Additional file 1: Table S4) which is absent from **Spn**-**VI** (Figure 5). Instead, the shortest intermolecular contact of the aniline **H2'** site in **Spn**-**VI** is of the **H2'**∙∙∙**A3** type and significantly longer than would be expected for an N − H∙∙∙O bond (the H∙∙∙O and N∙∙∙O distances are 2.71 and 3.38 Å, respectively), and the A and B molecules involved are of the opposite handedness.

**Figure 6:**
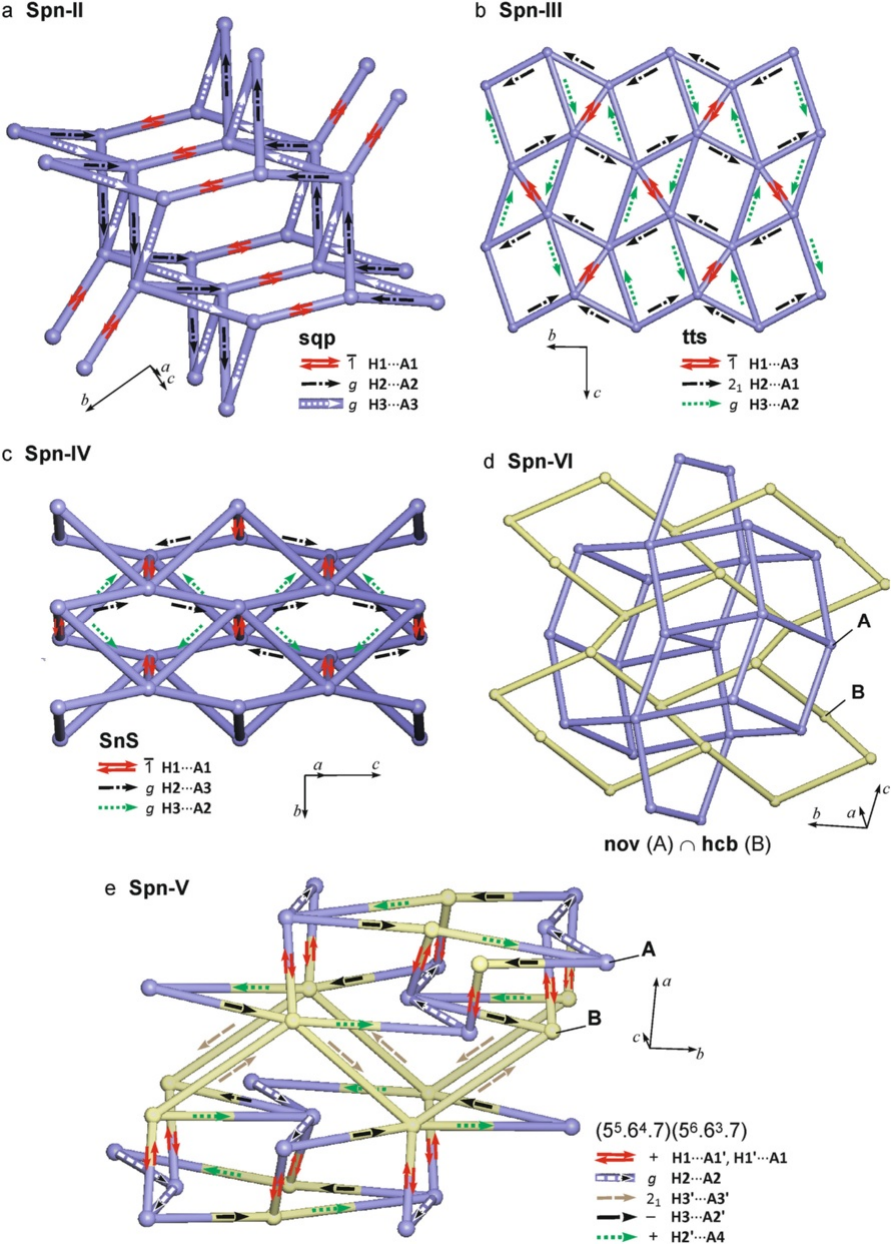
**Topology graphs for the hydrogen bonded structures of four polymorphs of sulfapyridine (Spn):** **a)** **the sqp framework of form II,** **b)** **the tts net of form III,** **c)** **the SnS-type net of form IV,** **d)** **interpenetration of the nov-type framework (A molecules) by an hcb-type layer (B molecules) and** **e)** **the framework of type V.**

The absence of the weak **H2'**∙∙∙**A4** connection in **Spn**-**VI** may carry a penalty in stabilisation energy but may permit the larger **Spn** molecules to adopt the same 3D packing arrangement as those of **Stz**. The interpenetration of the H-bonded framework of A molecules by the layers of B molecules in **Spn**-**VI** (Figure 6d) is described by the symbol F6_5_[4^4^.6^6^-**nov**]:[*g*.2_1_.*g*.2_1_.${\overline{1}}^{\mathrm{II}}$] ∩ L4_3_[6^3^-**hcb**]:[2_1_.${\overline{1}}^{\mathrm{II}}$.2_1_] (short: F6_5_[4^4^.6^6^-**nov**] ∩ L4_3_[6^3^-**hcb**]). For completeness, the graphical and symbolic representations and connectivity tables for four other known polymorphs of **Spn** are given in Figure 6, Table 1 and Figure 2, respectively, and details of the H-bonded structures the assignment of H and A sites are given in the Additional file 1.

## Discussion

### Relationships between the Stz polymorphs IV, V and III

The topology graphs and associated chemical and symmetry information for each of **Stz**-**IV**, **Stz**-**V** and **Stz**-**III** in Figure 3a, b and c immediately reveal the following relationships:

1. An **sql** net is formed in each case. Note that the three nets are drawn with their actual geometry and in matching orientations when strictly the depiction of the correct connectivity between the nodes would be sufficient, for example in a standard square grid.
2. The H-bonded structures of **Stz**-**IV** and **Stz**-**V** are based on the same type (in terms of topology, chemistry and symmetry) of two-point hydrogen bond connection but they differ in the symmetry and chemistry of their one-point connections, which are however equivalent with respect to their topology and directionality.
3. The two independent molecules (A, B) of **Stz**-**III** are both four-connected nodes within a **sql** net. There are no hydrogen bonds of the A∙∙∙A or B∙∙∙B types. The A and B nodes agree completely in their chemistry and in the symmetry operation associated with their respective two-point connections However, the one-point connections differ chemically and in the associated symmetry elements, but not in their directionality. In the two single H-bond interactions of **Stz**-**III**, molecule A adopts the function of the H-bond donor of form **Stz**-**V** in one case and the acceptor function of **Stz**-**IV** in the other, with opposite functions provided by molecule B in each case. With this information, one can establish that, with regard to the type and orientation of the hydrogen bonds and handedness of the molecules involved, the **sql** net of **Stz**-**III** consists of alternating ladder fragments of the **Stz**-**IV** and **Stz**-**V** types.
4. With additional local symmetry information established in a previous study [38], it becomes clear that the correspondence of the A∙∙∙B and B∙∙∙A interactions in the H-bonded layer of **Stz**-**III** with the H-bonds in **Stz**-**V** and **Stz**-**IV**, respectively, even extends to their (local) symmetry.

Thus, the correct relationships between the H-bonded structures **Stz**-**III**, **Stz**-**V** and **Stz**-**IV** can be established readily with the proposed method. By contrast, it would be very difficult if not impossible to deduce these relationships from the conventional graph-set analysis of the corresponding three HBSs provided in section 5 of the Additional file 1.

The information obtained from the topology graphs is consistent and complementary with the results of a previous packing analysis [38] showing that **Stz**-**III** has a molecular bilayer in common with each of **Stz**-**IV** and **Stz**-**V**. These two types of double layer are just stacks of the H-bonded ladder fragments within the **sql** net which **Stz**-**III** has in common with **Stz**-**IV** and **Stz**-**V (**Figure 3a, b and c). Accordingly, **Stz**-**IV** and **Stz**-**V** have a molecular monolayer in common. This is a stack of simple chain fragments which is based on a two-point connection and forms part of their respective HBS.

In the connectivity table for **Stz**-**III** (Figure 2), the A∙∙∙B quadrant corresponds with **Stz**-**V** and the B∙∙∙A quadrant with the interactions of **Stz**-**IV**. The A∙∙∙A and B∙∙∙B quadrants are empty, which is in consistent with the assertions in point 3 above. The number of H-bonds formed by an individual molecule can be deduced from this connectivity table but not the number of neighbours involved in these interactions or the type of the resulting net.

**Figure 7:**
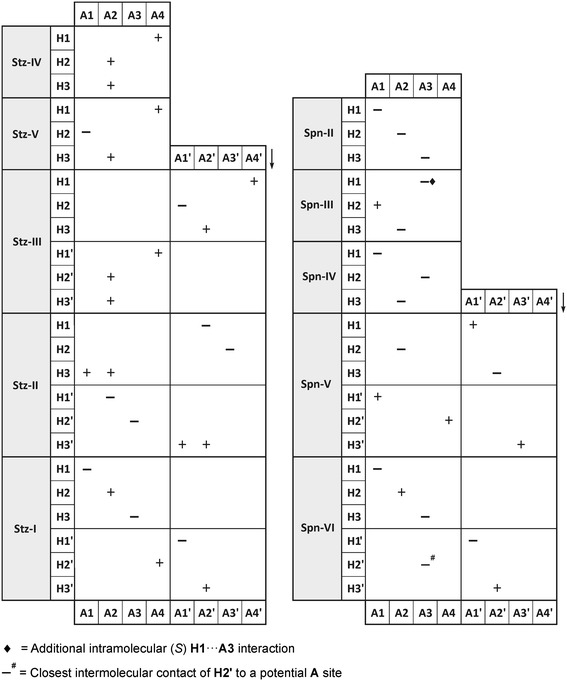
**Connectivity tables for the D − H∙∙∙A interactions in polymorphic forms of sulfathiazole (Stz) and sulfapyridine (Spn), with the symbols + and − indicating connections between molecules of the same or of the opposite handedness, respectively.**

Figure 7 shows an alternative version of the connectivity tables of Figure 2, in which symmetry elements are replaced by symbols for handedness relations. These still reflect similarities between HBSs, albeit on a lower level. For example, the configuration of plus and minus symbols in the tables for **Stz**-**III**, −**IV** and -**V** reflects also their complex relationships discussed above. Likewise, matching entries in the tables for **Stz**-**I** and **Spn**-**VI** reflect the similarity of their HBSs. The alternative connectivity table for **Stz**-**IV** contains exclusively plus symbols, indicating that its HBS consists of homochiral molecules. On the other hand, the absence of plus signs in the tables for **Spn**-**II** and **Spn**-**IV** indicates that all H-bonds in these polymorphs connect molecules of the opposite handedness.

### Comparison of the HBSs in polymorphs of Stz and Spn

The topology graphs of the separate **nov** and **hcb** nets of **Spn**-**VI** (not shown) are in complete agreement with those of **Stz**-**I**. The very close relationship between **Stz**-**I** and **Spn**-**VI**, which is also consistent with an earlier packing comparison, is also reflected in their connectivity tables and HBS symbols (Table 1 and Figure 2).

Four- (**Stz**-**III**, −**IV**, −**V**), five- (**Spn**-**II**, −**III**, −**IV**, −**V**) or six-connected (**Stz**-**II**) nets are formed, with the exception of **Stz**-**I** (4,6-connected) and **Spn**-**VI** (3,5-connected). There are four framework structures (**Stz**-**I**, **Spn**-**III**, −**IV**, −**VI**) and six layer structures. Overall, the connectivity tables in Figure 2 indicate that **Spn** has a general preference for the formation of **D1**∙∙∙**A1** interactions (four forms) which in all cases but one (**Spn**-**V**) result in a centrosymmetric dimer unit. However, there is only one such example (**Stz**-**I**) in the **Stz** family. Each HBS contains at least one interaction of the (**D2** or **D3**)∙∙∙(**A2** or **A3**) type involving an H atom if the NH_2_ group and a sulfonyl oxygen site. The **A4** position is engaged in H-bonding only in **Stz**-**I** and in the three closely related HBS of **Stz**-**III**, −**IV** and -**V**.

## Conclusions

The objective to compare different HBSs and to identify relationships between them has led to a graphical solution which combines established concepts (i.e. the interpretation of an HBS as a net, determination and classification of topology) with specific characteristics of HBSs (a link is defined by one or more H-bonds, all of which possess a chemical identity as well as directionality; a homomolecular link is associated with a handedness relationship/symmetry operation; differentiation between nodes that are topologically equivalent but crystallographically distinct). By comparison, only selected information about an HBS can be deduced from the proposed HBS symbol (its topology and specific characteristics of nodes) and connectivity table (the chemical identity of all H-bonds) representation. The former is intended as a general HBS descriptor in printed texts while the latter facilitates the comparison of the connections present in different HBSs that are based on matching H-bond donor and acceptor functional groups.

Ultimately, the usefulness of the proposed methodologies will have to be tested by applying them to other sets of crystal structures, and this will also provide pointers to necessary adjustments of their setup. The examples in this report demonstrate that HBS analysis and the identification of packing similarity based on geometrical methods are complementary. We intend to explore this topic further with an analysis of the more than 100 solvate structures of sulfathiazole.

## Experimental

### Crystal structure data

Crystal structure data from the Cambridge Structural Database [45] were used throughout (for details, see Additional file 1: Table S1). However, in the case of **Spn**-**IV** and **Spn**-**V** the HBS analysis was carried out with recalculated idealised positions of the NH_2_ hydrogen atoms, and in the case of **Spn**-**IV** the NH hydrogen atom was also recalculated (for details, see sections 3.5 and 3.6 of the Additional file 1). Details of the H-bonds defining the HBSs are collected in Additional file 1: Tables S4–S13).

### Determination, classification and visualisation of topology

The topologies of HBSs were determined and classified with the programs ADS and IsoTest of the TOPOS package [31] in the manner described by Barburin & Blatov [32]. The topology graphs for HBSs (Figures 3, 4 and 6) are based on nets drawn with the IsoCryst program of the TOPOS package [31].

### XPac studies

Structure comparisons [11] and the calculation of the dissimilarity index [7] were carried out in the previously described manner. All comparisons involving exclusively polymorphs of either Stz or Spn were based on geometrical parameters derived from the complete sets of non-H atomic positions. For comparisons between Stz and Spn, the atomic positions of the thiazole (Stz) or pyridine (Spn) ring were not used except for the carbon atom bonded to the sulfonamido N atom. Further details are given in section 4 of the Additional file 1.

## Endnote

^a^In the case of a common 2D net (**sql**, **hcb**, **hxl**) the RCSR symbol alone would be sufficient. For consistency, the point symbol is included for all examples discussed in this paper.

## Additional file

Additional file 1:
**Electronic Additional file** **1** **is available, consisting of a) crystal structure data, b) details of the assignment of** **H** **and** **A** **sites, c) geometrical parameters of hydrogen bonds, d) details of XPac studies, e) the graph-set description for** **Stz-** **III, −** **IV** **and** **-** **V.**

## Abbreviations

A: Hydrogen-bond acceptor
D − **H**: Hydrogen-bond donor group
HBS(s): Hydrogen-bonded structure(s)
RCSR: Reticular Chemistry Structure Resource
Spn: Sulfapyridine
Stz: Sulfathiazole
