# Supplementary material for: Describing hydrogen-bonded structures; topology graphs, nodal symbols and connectivity tables, exemplified by five polymorphs of each of sulfathiazole and sulfapyridine
Source: Chem Cent J. 2015 Jan 21;9:1. doi: 10.1186/s13065-014-0076-x (PMC4309923; doi:10.1186/s13065-014-0076-x)
Supplement: Additional file 1: — Electronic Additional file 1 is available, consisting of a) crystal structure data, b) details of the assignment of H and A sites, c) geometrical parameters of hydrogen bonds, d) details of XPac studies, e) the graph-set description for Stz- III, − IV and - V. [file 13065_2014_76_MOESM1_ESM.docx]

**Additional file 1**

**Describing hydrogen-bonded structures; topology graphs, nodal symbols and connectivity tables, exemplified by five polymorphs of each of sulfathiazole and sulfapyridine**

Michael B. Hursthouse^1^*

Email: [m.b.hursthouse@soton.ac.uk](mailto:m.b.hursthouse@soton.ac.uk)

David S. Hughes^1^

Email: [d.hughes](mailto:d.hughes)@soton.ac.uk

Thomas Gelbrich^2^

Email: [thomas.gelbrich@uibk.ac.at](mailto:thomas.gelbrich@uibk.ac.at)

Terence L. Threlfall^1^

Email: [t.threlfall@soton.ac.uk](mailto:t.threlfall@soton.ac.uk)

^1^Chemistry, Faculty of Natural and Environmental Sciences, University of Southampton, Southampton SO17 1BJ, UK

^2^Institut of Pharmacy, University of Innsbruck, Innrain 52, 6020, Austria

*Corresponding author

Contents

[1. Crystal structure data 3](#_Toc397547415Toc397616440)

[2. Assignment of corresponding H and A sites 4](#_Toc397547416Toc397616441)

[3. Geometrical parameters of D−H∙∙∙A bonds 6](#_Toc397547417Toc397616442)

[3.1. Stz-I 6](#_Toc397547418Toc397616443)

[3.2. Spn-VI 6](#_Toc397547419Toc397616444)

[3.3. Stz-II 7](#_Toc397547420Toc397616445)

[3.4. Stz-III 7](#_Toc397547421Toc397616446)

[3.5. Stz-IV 8](#_Toc397547422Toc397616447)

[3.6. Stz-V 8](#_Toc397547423Toc397616448)

[3.7. Spn-II 8](#_Toc397547424Toc397616449)

[3.8. Spn-III 9](#_Toc397547425Toc397616450)

[3.9. Spn-IV 9](#_Toc397547426Toc397616451)

[3.10. Spn-V 10](#_Toc397547427Toc397616452)

[4. Details of XPac studies 11](#_Toc397547428Toc397616453)

[4.1. Stz-IV, Stz-IV and Stz-III 11](#_Toc397547429Toc397616454)

[4.2. Stz-I / Spn-VI 11](#_Toc397547430Toc397616455)

[5. Graph-set description 13](#_Toc397547431Toc397616456)

[6. References 14](#_Toc397547432Toc397616457)

# Crystal structure data

#

**Table S1.** Crystal structure data of sulfathiazole (Stz) and sulfapyridine (Spn) used in this report

| Compound-Form | |  | Space group | CSD | Ref. |
| --- | --- | --- | --- | --- | --- |
|  |  | |  |  |  |
|  | **Stz-I** | | *P*2_1_/*c*, Z’ = 2 | SUTHAZ16 | [1] |
|  | **Stz-II** | | *P*2_1_/*n*, Z’ = 2 | SUTHAZ05 | [2] |
|  | **Stz-III** | | *P*2_1_/*c*, Z’ = 2 | SUTHAZ17 | [1] |
|  | **Stz-IV** | | *P*2_1_/*c*, Z’ = 1 | SUTHAZ18 | [1] |
|  | **Stz-V** | | *P*2_1_/*n*, Z’ = 1 | SUTHAZ19 | [1] |
|  |  | |  |  |  |
|  |  | |  |  |  |
|  | **Spn-II** | | *P*2_1_/*c*, Z’ = 1 | BEWKUJ11 | [3] |
|  | **Spn-III** | | *C*2/*c*, Z’ = 1 | BEWKUJ12 | [3] |
|  | **Spn-IV** | | *P*2_1_/*c*, Z’ = 1 | BEWKUJ05 | [4] |
|  | **Spn-V** | | *Pbca*, Z’ = 2 | BEWKUJ13 | [3] |
|  | **Spn-VI** | | *P*2_1_/*n*, Z’ = 2 | BEWKUJ14 | [5] |
|  |  | |  |  |  |

# Assignment of corresponding H and A sites

**
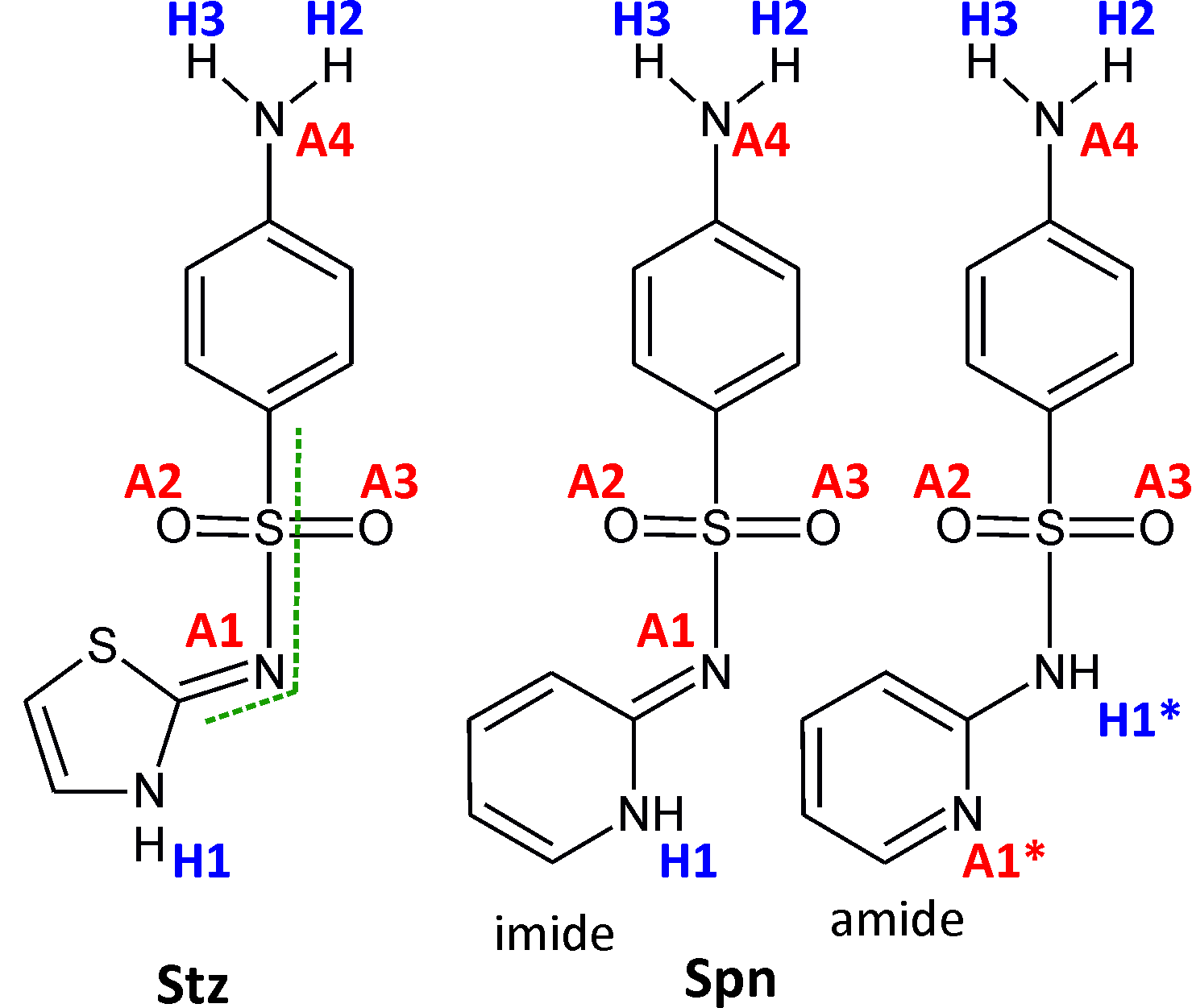
**

Fig. S1 Definition of H and A sites in the molecules of sulfathiazole (Stz; broken line: torsion angle C−N−S−C) and sulfapyridine (Spn).

Definition of matching **H** and **A** sites (see Figure S1):

1. **H1** is the H atom of the amido nitrogen NH group

2. **H2** is the H atom of the aniline NH2 group which gives the largest absolute value of the pseudo-torsion angle **A2**−S∙∙∙N1−H and **H3** is the other H atom of the same group.

3. **A1** is the imido N atom.

4. **A2** is the sulfonyl O atom associated with the largest absolute value of the torsion angle C−N−S−O and **A3** is the other sulfonyl O atom.

5. **A4** is the aniline N atom.

**Table S2.** Assignment of corresponding **H** and **A** functions in the polymorphs **I** − **V** of **Stz** and **II** − **VI** of **Spn**

| Compound-Form | |  | Molecule | **H1** | **H2** | **H3** | **A1** | **A2** | **A3** | **A4** |
| --- | --- | --- | --- | --- | --- | --- | --- | --- | --- | --- |
|  | **Stz-I** | | A | H7 | H1 | H2 | N2 | O1 | O2 | N1 |
|  | **Stz-I** | | B (‘) | H16 | H10 | H11 | N5 | O3 | O4 | N4 |
|  | **Stz-II** | | A | H3 | H2 | H1 | N2 | O1 | O2 | N1 |
|  | **Stz-II** | | B (‘) | H12 | H10 | H11 | N5 | O4 | O3 | N4 |
|  | **Stz-III** | | A | H7 | H1 | H2 | N2 | O1 | O2 | N1 |
|  | **Stz-III** | | B (‘) | H16 | H10 | H11 | N5 | O3 | O4 | N4 |
|  | **Stz-IV** | |  | H7 | H1 | H2 | N2 | O1 | O2 | N1 |
|  | **Stz-V** | |  | H7 | H1 | H2 | N2 | O1 | O2 | N1 |
|  |  | |  |  |  |  |  |  |  |  |
|  |  | |  |  |  |  |  |  |  |  |
|  | **Spn-II** | |  | H7 | H2 | H1 | N1 | O2 | O1 | N2 |
|  | **Spn-III** | |  | H6 | H5 | H4 | N1 | O1 | O2 | N2 |
|  | **Spn-IV** | |  | H3^#^ | H2A^#^ | H2B^#^ | N1 | O2 | O1 | N2 |
|  | **Spn-V** | | A | H7 | H2A^#^ | H2B^#^ | N1 | O2 | O1 | N2 |
|  | **Spn-V** | | B (‘) | H18 | H5A^#^ | H5B^#^ | N4 | O3 | O4 | N5 |
|  | **Spn-VI** | | A | H1 | H2 | H3 | N1 | O2 | O1 | N3 |
|  | **Spn-VI** | | B (‘) | H12(*) | H14 | H13 | N5(*) | O4 | O3 | N6 |
|  |  | |  |  |  |  |  |  |  |  |

^#^ = Simulated H atoms in idealised positions

**Table S3.** Torsion angles used for the definition of corresponding **A** and **H** sites and the torsion angle C−N−S−C used for the analysis of pseudo-chirality relationships between independent molecules

| Compound- Form | |  | Molecule | C−N−S−**A2** | C−N−S−**A3** | **A2**−S∙∙∙N−**H2** | **A2**−S∙∙∙N−**H3** | C−N−S−C |
| --- | --- | --- | --- | --- | --- | --- | --- | --- |
|  |  | |  |  |  |  |  |  |
|  | **Stz-I** | | A | -161.2 | -33.3 | 115.2 | -28.9 | 84.9 |
|  | **Stz-I** | | B | 167.0 | 39.3 | -138.9 | 26.6 | -78.9 |
|  | **Stz-II** | | A | -145.4 | -17.6 | 118.2 | -60.2 | 99.6 |
|  | **Stz-II** | | B | -164.4 | -37.1 | 97.1 | -37.0 | 81.1 |
|  | **Stz-III** | | A | -168.9 | -40.0 | 140.7 | -102.0 | 77.8 |
|  | **Stz-III** | | B | -166.3 | -36.5 | 127.0 | -103.8 | 80.2 |
|  | **Stz-IV** | |  | -168.2 | -39.2 | 130.5 | -102.9 | 78.3 |
|  | **Stz-V** | |  | -166.9 | -37.2 | 134.2 | -102.4 | 79.6 |
|  |  | |  |  |  |  |  |  |
|  |  | |  |  |  |  |  |  |
|  | **Spn-II** | |  | 167.4 | 39.4 | 149.5 | 61.4 | -78.8 |
|  | **Spn-III** | |  | -173.2 | -45.2 | 158.1 | -44.5 | 72.2 |
|  | **Spn-IV** | |  | 164.9 | 37.0 | -164.4 | -73.7 | 80.1 |
|  | **Spn-V** | | A | -176.4 | -48.4 | -164.0 | -46.3 | 68.9 |
|  | **Spn-V** | | B | 179.9 | 52.2 | 153.3 | -65.2 | -65.5 |
|  | **Spn-VI** | | A | -174.7 | -48.7 | 120.9 | -68.0 | 70.4 |
|  | **Spn-VI** | | B | 177.6 | -54.7 | 154.6 | -26.7 | 61.9 |
|  |  | |  |  |  |  |  |  |

# Geometrical parameters of D−H∙∙∙A bonds

## Stz-I

**Table S4.** Intermolecular hydrogen bonds in the crystal structure **of Stz-I** (calculated with the data of SUTHAZ16 [1])

| Type | Symm. | D−**H**∙∙∙**A** | D−**H** | **H**∙∙∙**A** | D∙∙∙**A** | ∠D−**H**∙∙∙**A** |
| --- | --- | --- | --- | --- | --- | --- |
|  |  |  |  |  |  |  |
| **H1**∙∙∙**A1** | $\bar{1}$ | N3−H7∙∙∙N2^iii^ | 0.84(3) | 2.05(3) | 2.883(3) | 167(3) |
| **H2**∙∙∙**A2** | 2_1_ | N1−H1∙∙∙O1^i^ | 0.95(4) | 2.02(3) | 2.951(3) | 166(3) |
| **H3**∙∙∙**A3** | *g* | N1−H2∙∙∙O2^ii^ | 0.84(3) | 2.33(3) | 2.955(3) | 131(2) |
| **H1’**∙∙∙**A1’** | $\bar{1}$ | N6−H16∙∙∙N5^vi^ | 0.88(3) | 2.00(3) | 2.867(3) | 169(2) |
| **H3’**∙∙∙**A2’** | 2_1_ | N4−H11∙∙∙O3^v^ | 0.88(3) | 2.36(3) | 3.095(3) | 141(2) |
| **H2’**∙∙∙**A4** | + | N4−H10∙∙∙N1^iv^ | 0.94(3) | 2.29(3) | 3.221(4) | 171(2) |
| Symmetry operations: (i) 1-x,1/2+y,-1/2-z (ii) x,3/2-y,-1/2+z (iii) 2-x,1-y,-z (iv) x,3/2-y,1/2+z (v) 2-x,1/2+y,1/2-z (vi) 2-x,-y,-z | | | | | | |

## Spn-VI

**Table S5.** Intermolecular hydrogen bonds in the crystal structure of **Spn-VI** (calculated with the data of BEWKUJ14 [5])

| Type | Symm. | D−**H**∙∙∙**A** | | D−**H** | **H**∙∙∙**A** | D∙∙∙**A** | ∠D−**H**∙∙∙**A** |
| --- | --- | --- | --- | --- | --- | --- | --- |
|  |  |  | |  |  |  |  |
| **H1**∙∙∙**A1** | $\bar{1}$ | N2−H1∙∙∙N1^i^ | | 0.84(3) | 2.09(3) | 2.929(4) | 178(3) |
| **H2**∙∙∙**A2** | 2_1_ | N3−H2∙∙∙O2^ii^ | | 0.85(3) | 2.17(3) | 3.013(4) | 171(3) |
| **H3**∙∙∙**A3** | *g* | N3−H3∙∙∙O1^iii^ | | 0.90(3) | 2.03(3) | 2.928(4) | 173(3) |
| **H1’***∙∙∙**A1’*** | $\bar{1}$ | N4−H12∙∙∙N5^iv^ | | 0.86(4) | 2.07(4) | 2.932(4) | 175(3) |
| **H3’**∙∙∙**A2’** | 2_1_ | N6−H13∙∙∙O4^v^ | | 0.84(3) | 2.46(3) | 3.186(4) | 145(3) |
| Closest contact between A and B molecules: | | | | | | | |
| **H2’**∙∙∙**A3** | − | | N6−H14∙∙∙O1^vi^ | 0.88(4) | 2.71(3) | 3.384(4) | 134(3) |
| Symmetry operations: (i) 2-x,2-y,-z (ii) 3/2-x,-1/2+y,1/2-z (iii) 1/2+x,3/2-y,1/2+z (iv) –x,1-y,-z (v) 1/2-x,1/2+y,1/2-z (vi) 1-x, 2-y, -z | | | | | | | |
|  | | | | | | | |

## Stz-II

**Table S6.** Intermolecular hydrogen bonds in the crystal structure of **Stz-II** (calculated with the data of SUTHAZ05 [2])

| Type | Symm. | D−**H**∙∙∙**A** | D−**H** | **H**∙∙∙**A** | D∙∙∙**A** | ∠D−**H**∙∙∙**A** |
| --- | --- | --- | --- | --- | --- | --- |
| **H3**∙∙∙**A1** | 2_1_ | N1−H1∙∙∙N2^i^ | 0.90(3) | 2.14(3) | 3.017(5) | 166(3) |
| **H3**∙∙∙**A2** | 2_1_ | N1−H1∙∙∙O1^i^ | 0.90(3) | 2.54(3) | 3.211(4) | 132(2) |
| **H2**∙∙∙**A3’** | − | N1−H2∙∙∙O3^ii^ | 0.94(4) | 2.09(4) | 3.010(4) | 167(4) |
| **H1**∙∙∙**A2’** | − | N3−H3∙∙∙O4^iii^ | 0.86 | 2.04 | 2.865(4) | 161 |
| **H2’**∙∙∙**A3** | − | N4−H10∙∙∙O2^iv^ | 0.91(2) | 2.24(2) | 3.061(4) | 151(2) |
| **H3’**∙∙∙**A2’** | 2_1_ | N4−H11∙∙∙O4^v^ | 0.90(3) | 2.36(3) | 3.117(4) | 142(3) |
| **H3’**∙∙∙**A1’** | 2_1_ | N4−H11∙∙∙N5^v^ | 0.90(3) | 2.44(3) | 3.267(4) | 154(3) |
| **H1’**∙∙∙**A2** | − | N6−H12∙∙∙O1^vi^ | 0.86 | 1.94 | 2.794(4) | 173 |
| Symmetry operations: (i) 1/2-x,1/2+y,-1/2-z (ii) –x,1-y,-z (iii) 1/2+x,1/2-y,-1/2+z (iv) –x,-y,-z (v) 1/2-x,-1/2+y,1/2-z (vi) 1/2+x,1/2-y,1/2+z | | | | | | |

## Stz-III

**Table S7.** Intermolecular hydrogen bonds in the crystal structure of **Stz-III** (calculated with the data of SUTHAZ17 [1])

| Type | Symm. | D−**H**∙∙∙**A** | D−**H** | **H**∙∙∙**A** | D∙∙∙**A** | ∠D−**H**∙∙∙**A** |
| --- | --- | --- | --- | --- | --- | --- |
| **H1**∙∙∙**A4'** | (2_1_) | N3−H7∙∙∙N4 | 0.88(2) | 1.98(3) | 2.846(4) | 167(3) |
| **H2**∙∙∙**A1'** | (*g*) | N1−H1∙∙∙N5^i^ | 0.88(2) | 2.33(2) | 3.184(4) | 162(3) |
| **H3**∙∙∙**A2'** | (2_1_) | N1−H2∙∙∙O3 | 0.885(19) | 2.140(18) | 3.001(4) | 164(3) |
| **H1'**∙∙∙**A4** | (2_1_) | N6−H16∙∙∙N1^ii^ | 0.89(3) | 2.02(3) | 2.899(4) | 171(4) |
| **H2'**∙∙∙**A2** | (*t*) | N4−H10∙∙∙O1^iii^ | 0.88(3) | 2.14(3) | 3.006(4) | 169(3) |
| **H3'**∙∙∙**A2** | (2_1_) | N4−H11∙∙∙O1^ii^ | 0.85(2) | 2.181(18) | 2.977(4) | 155(3) |
| Symmetry operations: (i) –x,1-y,-z (ii) x,-1+y,z (iii) 1-x,-1/2+y,1/2-z | | | | | | |
|  | | | | | | |

## Stz-IV

**Table S8.** Intermolecular hydrogen bonds in the crystal structure of **Stz-IV** (calculated with the data of SUTHAZ18 [1])

| Type | Symm. | D−**H**∙∙∙**A** | D−**H** | **H**∙∙∙**A** | D∙∙∙**A** | ∠D−**H**∙∙∙**A** |
| --- | --- | --- | --- | --- | --- | --- |
|  |  |  |  |  |  |  |
| **H1**∙∙∙**A4** | 2_1_ | N3−H7∙∙∙N1^i^ | 0.89(2) | 1.98(2) | 2.845(2) | 166(2) |
| **H2**∙∙∙**A2** | *t* | N1−H1∙∙∙O1^ii^ | 0.89(2) | 2.13(2) | 3.001(2) | 165(2) |
| **H3**∙∙∙**A2** | 2_1_ | N1−H2∙∙∙O1^i^ | 0.85(3) | 2.19(3) | 2.989(2) | 158(2) |
| Symmetry operations: (i) 2-x,1/2+y,3/2-z (ii) 1+x,y,z | | | | | | |

## Stz-V

**Table S9.** Intermolecular hydrogen bonds **Stz-V** (calculated with the data of SUTHAZ19 [1])

| Type | Symm. | D−**H**∙∙∙**A** | D−**H** | **H**∙∙∙**A** | D∙∙∙**A** | ∠D−**H**∙∙∙**A** |
| --- | --- | --- | --- | --- | --- | --- |
|  |  |  |  |  |  |  |
| **H1**∙∙∙**A4** | 2_1_ | N3−H7∙∙∙N1^i^ | 0.86(2) | 2.06(2) | 2.902(3) | 166.2(18) |
| **H2**∙∙∙**A1** | *g* | N1−H1∙∙∙N2^ii^ | 0.89(2) | 2.36(2) | 3.173(2) | 153(2) |
| **H3**∙∙∙**A2** | 2_1_ | N1−H2∙∙∙O1^i^ | 0.83(3) | 2.19(3) | 2.988(2) | 160(2) |
| Symmetry operations: (i) 3/2-x,1/2+y,1/2-z (ii) 1/2+x,1/2-y,-1/2+z | | | | | | |
|  | | | | | | |

## Spn-II

**Table S10.** Intermolecular hydrogen bonds in the crystal structure of **Spn-II** (calculated with the data of BEWKUJ11 [3])

| Type | Symm. | D−**H**∙∙∙**A** | D−**H** | **H**∙∙∙**A** | D∙∙∙**A** | ∠D−**H**∙∙∙**A** |
| --- | --- | --- | --- | --- | --- | --- |
| **H1**∙∙∙**A1** | $\bar{1}$ | N3−H7∙∙∙N1^i^ | 1.01 | 1.90 | 2.914(4) | 174 |
| **H2**∙∙∙**A2** | *g* | N2−H2∙∙∙O2^ii^ | 1.02 | 2.18 | 3.069(5) | 145 |
| **H3**∙∙∙**A3** | *g* | N2−H1∙∙∙O1^iii^ | 1.01 | 2.15 | 3.117(5) | 158 |
| Symmetry operations: (i) 2-x,2-y,1-z (ii) x,3/2-y,1/2+z (iii) 1+x,3/2-y,1/2+z | | | | | | |
|  | | | | | | |

## Spn-III

**Table S11.** Hydrogen bonds in the crystal structure of **Spn-III** (calculated with the data of BEWKUJ12 [3])

| Type | Symm. | D−**H**∙∙∙**A** | D−**H** | **H**∙∙∙**A** | D∙∙∙**A** | ∠D−**H**∙∙∙**A** | |
| --- | --- | --- | --- | --- | --- | --- | --- |
| **H1**∙∙∙**A3** | *S* (intra) | N3−H6∙∙∙O2 | 0.96(3) | 2.05(3) | 2.839(4) | 138(3) | |
| **H1**∙∙∙**A3** | $\bar{1}$ | N3−H6∙∙∙O2^i^ | 0.96(3) | 2.17(3) | 2.884(4) | 130(3) | |
| **H2**∙∙∙**A1** | 2_1_ | N2−H5∙∙∙N1^ii^ | 0.99(3) | 2.09(3) | 3.069(4) | 172(5) | |
| **H3**∙∙∙**A2** | *g* | N2−H4∙∙∙O1^iii^ | 0.96(3) | 2.06(3) | 3.000(4) | 166(4) | |
| Symmetry operations: (i) 1/2-x,3/2-y,-z (ii) 1/2-x,-1/2+y,1/2-z (iii) x,2-y,1/2+z | | | | | | | |
|  | | | | | | |  |

## Spn-IV

Approximate positions for the H atoms bonded to N2 and N3 have been calculated as follows (fractional coordinates x, y, z):

H2A 0.1171 0.5452 0.7809

H2B 0.1187 0.7608 0.7704

H3 0.5711 0.3850 0.5600

**Table S12**. Intermolecular hydrogen bonds in the crystal structure of Spn-IV (calculated with the data of BEWKUJ05 [4]) and with with the H atoms at N2 and N3 in idealised positions (^#^)

| Type | Symm. | D−**H**∙∙∙**A** | D−**H** | **H**∙∙∙**A** | D∙∙∙**A** | ∠D−**H**∙∙∙**A** |
| --- | --- | --- | --- | --- | --- | --- |
| **H1**∙∙∙**A1** | $\bar{1}$ | N3−H3^#^∙∙∙N1^i^ | 0.97^#^ | 1.94^#^ | 2.9095 | 180^#^ |
| **H2**∙∙∙**A3** | *g* | N2−H2A^#^∙∙∙O1^ii^ | 1.03^#^ | 2.05^#^ | 3.0742 | 180^#^ |
| **H3**∙∙∙**A2** | *g* | N2−H2B^#^∙∙∙O2^iii^ | 1.00^#^ | 2.00^#^ | 3.0053 | 180^#^ |
| Symmetry operations: (i) 1-x,1-y,1-z (ii) x,1/2-y,1/2+z (ii) x,3/2-y,1/2+z | | | | | | |
|  | | | | | | |

## Spn-V

Approximate positions for the H atoms bonded to N2 and N5 have been calculated as follows (fractional coordinates x, y, z):

H2A^#^ 0.5822 0.5708 -0.1449

H2B^#^ 0.5743 0.5148 -0.0242

H5A^#^ 0.5308 0.0157 0.3069

H5B^#^ 0.4810 0.0221 0.2442

**Table S13.** Intermolecular hydrogen bonds in the crystal structure of Spn-V (calculated with the data of BEWKUJ13 [3]) and with the H atoms at N2 and N5 in calculated positions (^#^)

| Type | Symm. | D−**H**∙∙∙**A** | D−**H** | **H**∙∙∙**A** | D∙∙∙**A** | ∠D−**H**∙∙∙**A** |
| --- | --- | --- | --- | --- | --- | --- |
| **H1**∙∙∙**A1’** | + | N3−H7∙∙∙N4^i^ | 1.05 | 1.83 | 2.8721 | 178 |
| **H2**∙∙∙**A2** | *g* | N2−H2A^#^∙∙∙O2^ii^ | 1.08^#^ | 2.16^#^ | 3.2355 | 180^#^ |
| **H3**∙∙∙**A2’** | − | N2−H2B^#^∙∙∙O3 | 0.98^#^ | 1.96^#^ | 2.9373 | 180^#^ |
| **H1’**∙∙∙**A1** | + | N6−H18∙∙∙N1 | 1.05 | 1.87 | 2.9049 | 168 |
| **H2’**∙∙∙**A4** | + | N5−H5A^#^∙∙∙N2^iii^ | 1.06^#^ | 2.11^#^ | 3.1716 | 180^#^ |
| **H3’**∙∙∙**A3’** | 2_1_ | N5−H5B^#^∙∙∙O4^iv^ | 1.00^#^ | 2.00^#^ | 2.9975 | 180^#^ |
| Symmetry operations: (i) 3/2-x,1/2+y,z (ii) x,3/2-y,-1/2+z (iv) 1-x,-1/2+y,1/2-z (iii) x,1/2-y,1/2+z | | | | | | |
|  | | | | | | |

# Details of XPac studies

All comparisons were carried out with the program XPac [6]. Dissimilarity parameters were calculated in the previously described manner [7] (see ref. [8] for additional reference examples).

## Stz-IV, Stz-IV and Stz-III

The XPac results relating to the packing relationships of these three polymorphs have been discussed in detail elsewhere [1]. Here we report additionally the dissimilarity indices *x* and distance parameters *d* for the various 2D layer relationships in this set. All calculations were based on geometrical parameters derived from the complete sets of 16 non-H atomic positions.

**Table S14.** Dissimilarity parameters *x* and *d* for XPac comparisons involving **Stz-III**, -**IV** and –**V** (Dim = dimensionality, SC = supramolecular construct).

| Structure 1 | Structure 2 | Dim | SC | *x* | *d* [Å] |
| --- | --- | --- | --- | --- | --- |
| **Stz**-**III** | **Stz**-**IV** | 2D | bilayer 1 | 1.7 | 0.06 |
| **Stz**-**III** | **Stz**-**V** | 2D | bilayer 2 | 1.7 | 0.08 |
| **Stz**-**IV** | **Stz**-**V** | 2D | monolayer | 1.3 | 0.05 |
| **Stz-III**,  **cluster A** | **Stz-III**,  **cluster B** | 2D | local symmetry, monolayer | 2.9 | 0.04 |

## Stz-I / Spn-VI

The 3D structural relationship between **Stz**-**I** and **Spn**-VI was previously discussed elsewhere [5]. Here we report additionally the dissimilarity index *x* and distance parameter *d*. This comparison was based on geometrical parameters that were obtained using 12 atomic positions, namely all non-H atomic positions apart from those of the thiazole (Stz) and pyridine (Spn) rings, but including their respective C atom bonded to the sulfonamido N atom.

**Table S15.** Dissimilarity parameters *x* and *d* for the XPac comparison between **Stz-I** and **Spn**-**VI** (Dim = dimensionality, SC = supramolecular construct).

| Structure 1 | Structure 2 | Dim. | SC | *x* | *d* [Å] |
| --- | --- | --- | --- | --- | --- |
| **Stz**-**I** | **Spn**-V**I** | 3D | packing similarity;  homoestructurality | 12.7 | 0.66 |

.

| **Table S16.** Corresponding lattice parameters of **Stz-I** and **Spn**-**VI** | | | | | |
| --- | --- | --- | --- | --- | --- |
|  | **Stz**-**I** | |  | **Spn**-V**I** | |
| *t*_1_ | $100$ | 10.534 Å |  | $100$ | 10.827 Å |
| *t*_2_ | $0\bar{1}0$ | 12.936 Å |  | $010$ | 14.932 Å |
| *t*_3_ | $\bar{1}0\bar{1}$ | 17.203 Å |  | $001$ | 15.486 Å |
| ∠*t*_2,3_ |  | 90° |  |  | 90° |
| ∠*t*_1,3_ |  | 107.9° |  |  | 110.07° |
| ∠*t*_1,3_ |  | 90° |  |  | 90° |

# Graph-set description

**Table S17.** Second-level graph-set description according Etter [9-11] of the hydrogen bonded structures in three polymorphs of sulfathiazole, calculated with Mercury [12].

| **Stz-IV** | **Stz-IV** | **Stz-III** |
| --- | --- | --- |
|  |  |  |
| a = **H2**∙∙∙**A2** | a = **H2**∙∙∙**A1** | a = **H2**∙∙∙**A1'** |
| b = **H3**∙∙∙**A2** | b = **H3**∙∙∙**A2** | b = **H3**∙∙∙**A2'** |
| c = **H1**∙∙∙**A4** | c = **H1**∙∙∙**A4** | c = **H1**∙∙∙**A4'** |
|  |  | d = **H2'**∙∙∙**A2** |
|  |  | e = **H3'**∙∙∙**A2** |
|  |  | f = **H1'**∙∙∙**A4** |
|  |  |  |
| C1,1(8) a | C1,1(8) a | D1,1(2) a |
| C1,1(8) b | C1,1(8) b | D1,1(2) b |
| C1,1(10) c | C1,1(10) c | D1,1(2) c |
| C1,2(4) >a<b | C2,2(8) >b>c | D1,1(2) d |
| C2,2(8) >a>c | R2,2(18) >b<c | D1,1(2) e |
| C2,2(8) >b>c | R4,4(12) >a<b>a<b | D1,1(2) f |
| C2,2(16) >a>b | R4,4(12) >a>c>a>c | C1,2(4) >d<e |
| C2,2(18) >a<c | C4,4(22) >a>b<a<b | C2,2(8) >b>f |
| R2,2(18) >b<c | C4,4(24) >a>c<a<c | C2,2(8) >c>d |
| C3,4(20) >a>b<a<b | R4,4(32) >a>b>a>b | C2,2(8) >c>e |
| R3,4(20) >a>b>a<b | R4,4(36) >a<c>a<c | C2,2(16) >a>d |
| C4,4(26) >a>c<a<c | R6,6(38) >a>a>b>a>a<b | C2,2(16) >b>d |
| R4,4(26) >a>c>a<c | R6,6(38) >a>b>b<a>b>b | C2,2(16) >b>e |
| R5,6(36) >a>a>b>a>a<b | R6,6(40) >a>a>c>a>a<c | C2,2(18) >d<f |
| R5,6(36) >a>b>b<a<b<b | R6,6(44) >a>c>c<a>c>c | R2,2(18) >b<c |
| R6,6(42) >a>a>c>a>a<c |  | R2,2(18) >e<f |
| R6,6(46) >a>c>c<a<c<c |  | C2,2(20) >c>f |
|  |  | R4,4(12) >a<b>a<b |
|  |  | R4,4(12) >a>f>a>f |
|  |  | R4,4(32) >a>e>a>e |
|  |  | R4,4(36) >a<c>a<c |

# References

1. Gelbrich T, Hughes DS, Hursthouse MB, Threlfall TL: **Packing similarity in polymorphs of sulfathiazole.** *CrystEngComm* 2008, **10:**1328-1334.

2. Hughes DS, Hursthouse MB, Threlfall T, Tavener S: **A new polymorph of sulfathiazole.** *Acta Crystallogr, Sect C: Cryst Struct Commun* 1999, **55:**1831-1833.

3. Bar I, Bernstein J: **Conformational polymorphism VI: The crystal and molecular structures of form II, form III and form V of 4-amino-n-2-pyridinylbenzenesulfonamide (sulfapyridine).** *J Pharm Sci* 1985, **74:**255-263.

4. Bernstein J: **Polymorph iv of 4-amino-n-2-pyridinylbenzenesulfonamide (sulfapyridine).** *Acta Crystallogr, Sect C: Cryst Struct Commun* 1988, **44:**900-902.

5. Gelbrich T, Threlfall TL, Bingham AL, Hursthouse MB: **Polymorph VI of sulfapyridine: Interpenetrating two- and three-dimensional hydrogen-bonded nets formed from two tautomeric forms.** *Acta Crystallogr, Sect C: Cryst Struct Commun* 2007, **63:**o323-o326.

6. Gelbrich T, Hursthouse MB: **A versatile procedure for the identification, description and quantification of structural similarity in molecular crystals.** *CrystEngComm* 2005, **7:**324-336.

7. Gelbrich T, Threlfall TL, Hursthouse MB: ***XPac* dissimilarity parameters as quantitative descriptors of isostructurality: The case of fourteen 4,5'-substituted benzenesulfonamido-2-pyridines obtained by substituent interchange involving CF_3_/I/Br/Cl/F/Me/H** *CrystEngComm* 2012, **14:**5454-5464.

8. Gelbrich T, Threlfall TL, Hursthouse MB: **Eight isostructural 4,4'-disubstituted N-phenylbenzenesulfonamides.** *Acta Crystallogr, Sect C: Cryst Struct Commun* 2012, **68:**o421-o426.

9. Etter MC: **Encoding and decoding hydrogen-bond patterns of organic compounds.** *Acc Chem Res* 1990, **23:**120-126.

10. Etter MC, MacDonald JC, Bernstein J: **Graph-set analysis of hydrogen-bond patterns in organic crystals.** *Acta Crystallogr, Sect B: Struct Sci* 1990, **46:**256-262.

11. Bernstein J, Davis RE, Shimoni L, Chang N-L: **Patterns in hydrogen bonding: Functionality and graph set analysis in crystals.** *Angew Chem Int Ed* 1995, **34:**1555-1573.

12. Macrae CF, Bruno IJ, Chisholm JA, Edgington PR, McCabe P, Pidcock E, Rodriguez-Monge L, Taylor R, van de Streek J, Wood PA: **Mercury CSD 2.0 - new features for the visualization and investigation of crystal structures.** *J Appl Cryst* 2008, **41:**466-470.
